# Enterobius vermicularis Infestation: A Rare Cause of Appendicitis

**DOI:** 10.7759/cureus.78924

**Published:** 2025-02-12

**Authors:** Kugendran Ponniah, Suk Teen Larissa Yong, Dinesh Jayasooriya

**Affiliations:** 1 General Surgery, Northern Adelaide Local Health Network, Adelaide, AUS; 2 General Surgery, Royal Darwin Hospital, Darwin, AUS

**Keywords:** acute abdomen, enterobius vermicularis infestation, intestinal parasites, pediatric appendicitis, pinworm

## Abstract

*Enterobius vermicularis* (*E. vermicularis*, pinworm) is a common gastrointestinal parasite in children, occasionally implicated in appendiceal pathology. We report a case of a seven-year-old autistic male who presented with a three-day history of right iliac fossa pain, fever, nausea, and vomiting. Clinical and laboratory findings suggested acute appendicitis, prompting diagnostic laparoscopy and appendicectomy. Intraoperatively, a mildly inflamed appendix was noted with multiple mesenteric lymph nodes and visible roundworms at the appendiceal base, which were carefully suctioned out. Histopathology confirmed *E. vermicularis* infestation without definitive acute inflammatory changes. The patient had an uneventful recovery with postoperative anthelmintic therapy for the household. While the role of *E. vermicularis* in true appendicitis remains debated, its presence within the appendix can cause symptoms mimicking acute appendicitis. Given the diagnostic uncertainty and potential risk of appendiceal obstruction or secondary infection, appendicectomy remains a definitive and justified treatment in such cases, ensuring symptom resolution and preventing complications.

## Introduction

Acute appendicitis is a common pediatric surgical emergency, and while its etiology is often attributed to luminal obstruction, the role of *Enterobius vermicularis* (*E. vermicularis, *pinworm) in appendiceal pathology is increasingly recognized [1]. Though *E. vermicularis* is primarily associated with perianal pruritus, its presence in the appendix can lead to localized irritation, obstruction, and secondary bacterial infection, mimicking acute appendicitis [2]. Studies suggest that even in cases where histopathology does not confirm true inflammation, the presence of *E. vermicularis* may still contribute to the clinical presentation of appendicitis [3].

Given the diagnostic challenge posed by *E. vermicularis*-associated appendiceal disease, appendicectomy remains a justified intervention [4]. The overlap between parasitic infestation and true appendicitis means conservative management risks missing progressive inflammation or secondary infection cases. Additionally, surgical removal ensures complete resolution of symptoms, reduces the risk of recurrent pain, and allows for histopathological confirmation. Here, we present a case of a pediatric patient with appendiceal *E. vermicularis* infestation, reinforcing the role of appendicectomy as a definitive treatment in such presentations.

## Case presentation

A seven-year-old male child with autism and no significant surgical history presented to our hospital following transfer from a rural community in the Northern Territory. He had a three-day history of migraine and worsening right iliac fossa abdominal pain accompanied by fever, nausea, and vomiting. On examination, the child was febrile up to 39°C, and localized tenderness was noted in the right iliac fossa.

Laboratory results revealed an elevated white blood cell count of 13 × 10⁹/L and a C-reactive protein level of 32 mg/L, consistent with an inflammatory process. Given the clinical suspicion of appendicitis, the decision was made to proceed with diagnostic laparoscopy and appendicectomy.

**Figure 1:**
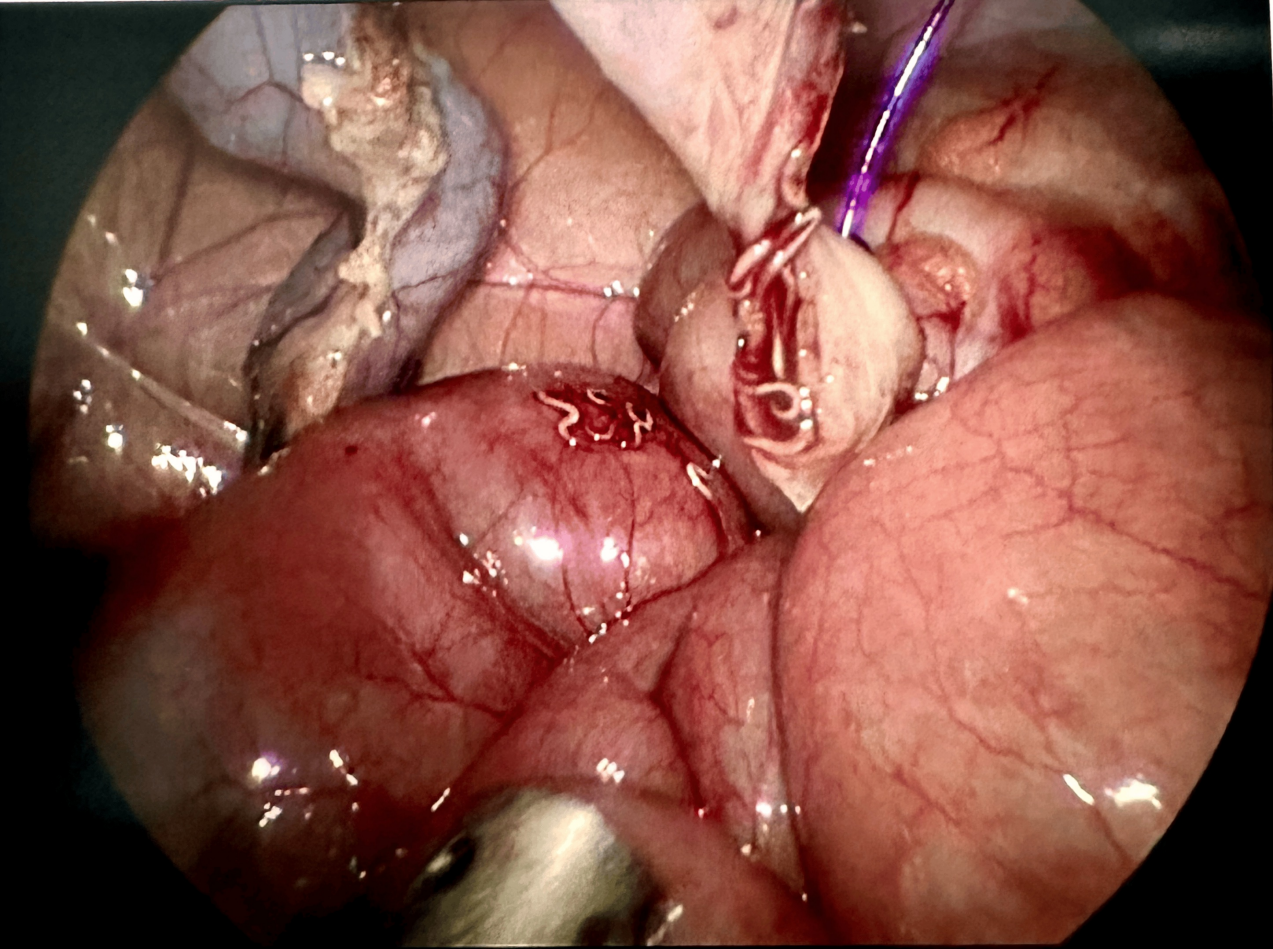
Enterobius vermicularis infestation in the appendiceal lumen

Intraoperative findings included a mildly injected and thickened appendix. Multiple enlarged mesenteric lymph nodes were noted, and during dissection of the appendix base, several roundworms were observed and carefully suctioned out (Figure 1). The appendix was removed without complication, and the pelvis was thoroughly examined to ensure no further parasites were present. Histopathological examination of the appendix confirmed a diagnosis of *E. vermicularis* infestation but without evidence of acute appendicitis.

The child recovered well postoperatively and was discharged the following day with complete resolution of symptoms following antihelmintic treatment with albendazole. A phone consultation for follow-up revealed an uneventful recovery.

This case highlights an unusual presentation of appendicitis in a pediatric patient with an underlying parasitic infestation. It serves as a reminder for clinicians to consider alternative causes of abdominal pain, especially in rural or endemic regions, and the importance of thorough intraoperative inspection when faced with atypical findings.

## Discussion

*E. vermicularis* is one of the most prevalent gastrointestinal helminth infections in the pediatric population [1]. While this parasite is primarily associated with perianal pruritus due to its characteristic nocturnal egg-laying behavior, it has also been implicated in various gastrointestinal pathologies, including appendiceal involvement. A recent meta-analysis has highlighted that *E. vermicularis* infection can mimic acute appendicitis, leading to diagnostic challenges. Among 100,000 cases reviewed, the incidence of confirmed parasitic involvement in appendiceal pathology was reported to be 2.9% [2].

The appendix is a frequent site for *E. vermicularis* colonization, with studies suggesting that the parasite is found within the lumen in approximately 90% of cases [3]. The exact pathophysiological mechanism by which *E. vermicularis* contributes to appendicitis remains uncertain. Still, it is hypothesized that the presence of the worms may lead to appendiceal obstruction, triggering localized inflammation and, in some cases, secondary bacterial infection [4]. This inflammatory response may then progress to classical symptoms of appendicitis, including right iliac fossa pain, nausea, vomiting, and anorexia. The pain may initially present as colicky and migratory in pediatric cases before localizing. There is also the possibility of late presentation, where the inflammation progresses to complications such as gangrenous appendicitis and perforation [5].

Interestingly, despite intraoperative findings suggestive of acute appendicitis, histopathological examination often does not confirm true acute inflammation of the appendix in cases associated with *E. vermicularis*. A study by Fleming et al. reported that only 30% of pediatric patients with pinworm-positive appendices exhibited histological features consistent with acute appendicitis [6]. This suggests that while the parasite may contribute to symptoms, its role in the direct pathogenesis of acute appendicitis remains complex and warrants further investigation.

From a surgical perspective, the identification of *E. vermicularis* during laparoscopic appendicectomy necessitates careful intraoperative management. Precise techniques should be employed because of the risk of contamination and potential postoperative complications, including intra-abdominal abscess formation. These include controlled suctioning, thorough peritoneal lavage, and avoiding unnecessary manipulation to prevent spillage of worms and eggs into the peritoneal cavity [7].

Although appendicectomy effectively alleviates the acute symptoms, it does not eradicate the underlying parasitic infection. Therefore, it is crucial to implement appropriate anthelmintics [8].

## Conclusions

*E. vermicularis* infestation can mimic acute appendicitis, creating diagnostic uncertainty. Although histopathological confirmation of acute inflammation is often lacking, the presence of the parasite in the appendix can lead to significant symptoms requiring intervention. Appendicectomy remains the most reliable treatment, providing definitive symptom relief and preventing potential complications. In cases of suspected appendicitis, surgical management is warranted to avoid the risks of delayed treatment.
